# Methods for external control groups for single arm trials or long‐term uncontrolled extensions to randomized clinical trials

**DOI:** 10.1002/pds.5141

**Published:** 2020-10-04

**Authors:** John D. Seeger, Kourtney J. Davis, Michelle R. Iannacone, Wei Zhou, Nancy Dreyer, Almut G. Winterstein, Nancy Santanello, Barry Gertz, Jesse A. Berlin

**Affiliations:** ^1^ Life Sciences Epidemiology, Optum Boston Massachusetts USA; ^2^ Global Epidemiology, Johnson & Johnson Titusville New Jersey USA; ^3^ Center for Biologics Evaluation and Research, U.S. Food and Drug Administration Silver Spring Maryland USA; ^4^ Pharmacoepidemiology, Merck & Co., Inc. Kenilworth New Jersey USA; ^5^ Real‐World Solutions, IQVIA Cambridge Massachusetts USA; ^6^ University of Florida Gainesville Florida USA; ^7^ Nancy Santanello Research Consultant Philadelphia Pennsylvania USA; ^8^ Blackstone Cambridge Massachusetts USA

**Keywords:** external control, long‐term extension (LTE), pharmacoepidemiology, real world data (RWD), single‐arm RCT

## Abstract

**Purpose:**

Clinical trials compare outcomes among patients receiving study treatment with comparators drawn from the same source. These internal controls are missing in single arm trials and from long‐term extensions (LTE) of trials including only the treatment arm. An external control group derived from a different setting is then required to assess safety or effectiveness.

**Methods:**

We present examples of external control groups that demonstrate some of the issues that arise and make recommendations to address them through careful assessment of the data source fitness for use, design, and analysis steps.

**Results:**

Inclusion and exclusion criteria and context that produce a trial population may result in trial patients with different clinical characteristics than are present in an external comparison group. If these differences affect the risk of outcomes, then a comparison of outcome occurrence will be confounded. Further, patients who continue into LTE may differ from those initially entering the trial due to treatment effects. Application of appropriate methods is needed to make valid inferences when such treatment or selection effects are present.

Outcome measures in a trial may be ascertained and defined differently from what can be obtained in an external comparison group. Differences in sensitivity and specificity for identification or measurement of study outcomes leads to information bias that can also invalidate inferences.

**Conclusion:**

This review concentrates on threats to the valid use of external control groups both in the scenarios of single arm trials and LTE of randomized controlled trials, along with methodological approaches to mitigate them.

**KEY POINTS**

- External controls may be needed to provide context to efficacy and/or safety in a trial that is single‐arm or becomes single‐arm (through a long‐term extension).
- Selection of an appropriate, fit‐for‐purpose data source is important.
- Both selection bias and information bias need to be addressed in the design and analysis.
- Historical examples are illustrative for key concepts and not exhaustive.
- Until guidance is codified in the countries of interest, health authority input in advance is useful.

## 1 BACKGROUND

Patient clinical experiences and outcomes are enormously complex and varied, which makes determining the effect of any treatment challenging.^1^ The structure imposed on patient care by a randomized, controlled trial (RCT) facilitates inferences about treatment effects,^2^, ^3^, ^4^ because it includes an internal comparison group (typically placebo or alternative active treatment) to show what patient response would have been without the study treatment. This internal control group is contemporaneously drawn from the same source as the treatment group and creates a benchmark of expected outcome occurrence against which the treated patients are compared. In the setting of a single arm trial, where all patients receive the treatment, or in a long‐term (usually open‐label) extension of an RCT, where participants are given the option to continue experimental treatment (or switch to it from the comparator) there is no built‐in control group.^5^, ^6^, ^7^ Oncology and treatments for rare diseases often employ single‐arm trials due to a reluctance to use a placebo and/or available patient population that limits sample size for a comparative study. Uncontrolled, long‐term extensions of an RCT may also be necessary due to what might be considered unethical long‐term use of placebo or discontinuation of effective treatment. In addition, the potential that any enrolled patient can eventually receive the study treatment in an LTE might provide an incentive for patients and investigators to participate. With either single‐arm trials or uncontrolled LTE, the use of an external control group may become necessary to assess safety or effectiveness by providing background rates for outcomes that can be appropriately compared with those for the study treatment in a way that informs decision‐making for regulators, providers and patients. In contrast to an internal control group that is drawn from the same population, an external control group is a group of patients that is external to the study, of similar disease severity (at equipoise) but who received a different treatment. The external control group can be patients treated at an earlier time (historical control) or patients treated contemporaneously, but in another setting.^6^ The pooled LTE of several tegaserod studies (Breakout Box #1) serves to illustrate how the need for an external control group can arise and the uncertainty that comes without such comparators.

Potential sources of data for external comparison groups include other trials, either historical or contemporary, or real‐world data (RWD). Patient‐level RWD sources can include protocol‐defined registries (disease, product, or geographic/institution‐based) and routinely‐collected administrative healthcare data, such as databases sourced from electronic medical records (EMR), health insurance claims, and linkages involving several of such sources (eg, claims or EMR linked to death certificates or a cancer registry).^8^ Each of these data sources, while potentially useful, must address both selection bias and information bias to serve as a basis for drawing valid comparisons and generating real‐world evidence (RWE).

A related term “synthetic controls” is sometimes used interchangeably with external control groups, for example in social sciences^9^ or studies of the population effect of vaccines.^10^ These methods create individual comparators for each member of a target group (such as treatment recipients in an RCT) as a weighted average of all potential members of the comparison group that best resembles the characteristics of the target group.^11^ Another definition of synthetic controls involves data generation for simulation studies that is based on actual data.^12^ Due to the potential for confusion across these uses and the connotation that data are partially fabricated, this paper uses the term external control group.

## 2 COMPARABILITY OF COHORTS

### 2.1 Selection bias

Identification of patients who would form a suitable external comparison group is not straightforward, and this is especially true for an untreated comparison group.^14^ The characteristics of patients who participate in a trial may differ considerably from those of trial‐eligible patients in the external data source, and may result in differences in outcome occurrence in the two groups. A framework of principles that can be used as a foundation for assessing the suitability of a potential comparison involves assessment of the proposed data source with respect to the three categories of variables used to derive effect measures: exposure, outcome, and other patient or medical care characteristics (covariates). The causal structure among these variables and the effect of conditioning on them through study design or analysis can create a range of biases that can be termed selection bias.^15^ We return, later in this paper, to exposure and outcome assessment, focusing for now on similarity of patient characteristics. Table 1 provides strengths and limitations associated with each of the data sources that might be considered for an external comparison group.

**TABLE 1:** Selected strengths and limitations of data sources for external comparison groups^16^

Data Source	Strengths	Limitations
Disease Registry	Pre‐specified data collection Good clinical detail regarding selected health outcomes Good disease ascertainment, including severity Often includes diverse patients and treatment settings Follow‐up period often longer than typical RCT Potential linkage to other sources for outcomes (eg, cancer, stroke, mortality, PROs from mobile apps)	Outcome measures may differ from trial Some covariates may not be available, for example, risk factors of special interest Details of non‐prescription and other medications may not be available Registry may not capture all outcomes of special interest. If follow‐up period is pre‐defined due to design or funding, this may limit assessment of longer‐term outcomes Potential for selection bias with regard to patients enrolled
Historical Clinical Trial	Good clinical detail Protocol‐specified care Similarity of trial exposure Similarity of trial covariate information collected Comparability of trial outcome measures May include placebo or placebo plus standard of care Equipoise at time of trial	Populations may differ substantially due to inclusion and exclusion criteria Historic standard of care may differ from the current trial context Non‐trial exposures may differ Definitions and ascertainment of patient characteristics and outcomes may differ Follow‐up time and censoring criteria may differ
Commercial Insurance or National Health Insurance Claims	Captures covered care regardless of site/provider type Many covariates available beyond indicated condition Good prescription medication details since medications represent billing for filled prescriptions Linkage to national registries (death, cancer, etc.) may be feasible or already present.	Outcomes ascertained differently; some outcomes may not be linked Only captures people with insurance No capture of medications administered during hospitalization No capture of non‐prescription medications or prescriptions paid outside insurance (eg, cash via discount cards) Limited lifestyle factors, laboratory values, and clinical detail on outcomes Data lag Capture may vary by age
Electronic Medical Records	Good disease ascertainment though partially in unstructured data Medications administered in hospital Medications prescribed in ambulatory care settings Patient‐reported medications include some non‐prescription (OTC) and may include actual usage (eg, PRN) Laboratory tests and results	Will not ascertain care received outside of provider network or specific EMR system unless the EMR come from an integrated health delivery system Does not capture prescription fills (primary non‐adherence) Inconsistent capture of other risk factors, concomitant medications, etc. Lack of standardization, across providers recording within and across EMR systems, complicates ascertainment of clinical characteristics and outcomes Outcome measures may differ from trial Data lag
Single Institution Medical Record Review	Useful for outcomes assessed during hospitalization and captured in medical record, including medical history and medications received during hospitalization	Labor‐intensive May lack comparable patients Less reliable for symptom‐based outcomes May not include outcomes that occur following discharge Patient selection concerns Patient consent may be needed for release if expedited approval is not sought/granted

*Note:* This table is not intended to be a complete list of strengths and limitations, but rather a selection of relevant ones. Further, there exists considerable heterogeneity among the specific examples of each type of data, so there are many exceptions to the general strengths and limitations presented here.

Breakout Box #1. **How an uncontrolled LTE study without an external comparison group led to uncertainty^13^**

Tegaserod, a 5HT_4_ receptor agonist, is effective for treating irritable bowel syndrome and is approved for this indication among women under age 65. Originally introduced in 2002, tegaserod was subsequently withdrawn for a cardiovascular safety signal (2007) and re‐introduced to the US market in 2019. Most of the studies within which tegaserod cardiovascular safety was evaluated were short, but seven RCTs provided LTE of their short‐term follow‐up among a total of 3289 tegaserod‐treated patients. These LTE studies provided an average of 227.3 days follow‐up compared to only 56.8 days in the controlled portion. During this additional LTE follow‐up, there were 4 cardiovascular events (3 coronary ischemic and 1 stroke). The coronary ischemic events occurred at days 122, 197, and 322 into the LTE, while the stroke occurred at 168 days. Although an incidence rate can be developed using these events and the person‐time that contributed to them, a suitable comparison incidence remained elusive.

External controls used: None.

Influence on decision‐making: Uncertain.

The effect of patient selection and its implications in terms of both generalizability (how similar are the studied patients to other populations, such as those observed in practice), and internal validity (how well the study addresses alternate explanations for the observed effect, in part arising from the similarity in characteristics between the treated and comparison groups) is evaluable to the extent tabulated patient characteristics reflect the relevant differences. An external comparison group should apply similar selection criteria for the participants in order to resemble the single arm or LTE treated group with respect to patient age, sex, clinical severity, and comorbidities. Mirroring the selection process of the trial will produce an external comparison group that shares characteristics with the treated group, but differences may remain due to inadequate specificity. In the blinatumomab example (Breakout Box #2), the same 18 years and older age criterion was applied both to trial participants and external comparators, yet the trial participants tended to be older (mean of 41 years, with 28% being 55+ years) than the external comparison group members (mean of 38 years, with 10% being 55+ years). Since the study outcomes included mortality, an imbalance on age would confound the comparison (due to it being a predictor of mortality) unless appropriate adjustment is made. Thus, having similar selection criteria may not be sufficient to balance compared groups across patient characteristics that might affect outcomes, so a method capable of balancing several variables simultaneously (such as propensity scores) can be useful.

Patient characteristics explicitly listed as criteria for inclusion or exclusion can be tabulated to show comparability between trial participants and an external comparison group. However, certain selection criteria might not be feasible to implement in the same manner in an external comparison group. Existing data sources from which external comparison groups are drawn may not record trial‐specific selection criteria in the same way or the variable may not be captured at all if the measure is not part of routine care (eg, specialized laboratory testing). Further, some criteria not explicitly listed may be part of the selection process, including ease of access to clinical care, adequate social support, willingness to participate in research, geographic or country‐specific differences, and physician‐assessed diagnosis, all of which are factors that could affect the risk of study outcomes and result in confounding if not addressed.

The setting of LTE involves some additional challenges. Patients enrolling in a single‐arm trial are new users, whereas those enrolling into an uncontrolled LTE are “survivors” of the initial treatment; they are patients who have experienced improvements in disease or at least did not experience adverse effects that diminished their willingness to continue treatment. There may also be new users in the LTE, since patients who had been assigned to the comparator group (placebo or other) typically cross over to the LTE treatment. This introduces an additional source of complexity, in that new users might experience more adverse events than continuers of the LTE treatment. These design challenges can often be addressed by following some basic design principles that are outlined elsewhere.^17^

### 2.2 Addressing selection bias

Differences that remain with respect to patient characteristics in the clinical trial participants and those of the external comparison group will need to be addressed to achieve balance on characteristics that may be prognostic of study outcomes. Standard observational research techniques may be applied including restriction, stratification, matching, modeling, and weighting. To match external controls to trial participants, a pool of potential participants in and external comparator group can be screened with respect to individual patient characteristics (age, sex, comorbidities, etc.) to find patients that are similar. However, such exhaustive matching will often not be feasible, since suitable comparison patients that match on all of the characteristics may not exist even within a large pool of comparator candidates (such as might arise from administrative data sources). Accordingly, one approach that has gained widespread acceptance, is exposure propensity scoring, which may be used in a variety of ways (eg, matching) to overcome this problem with multidimensionality. The trial participants and eligible patients from the external data source are assembled into a single dataset that contains variables for each of the characteristics and comorbidities considered important to balance, along with an “exposure” indicator of whether the patient came from the trial or the external data source. The propensity score is estimated using standard logistic regression approaches^20^ with the “outcome” variable being the data source (which is the exposure indicator) and the predictors being the set of variables to be balanced across exposure groups. This model provides the predicted probability of being in the trial cohort relative to the external cohort as a function of the covariates (the propensity score). This propensity score can be used to match, weight, adjust, or stratify. The blinatumomab example presented two sets of results, one based on risk factor stratification and standardization, and a subsequent approach that applied propensity score weighting (IPTW) with quite similar results. Stratification allowed easy visual assessment of the relevant comparisons between treatment groups within similar groups of patients (as defined by the stratification variables). Approximately 1 year after the results from the external comparison group for blinatumomab were published, results from a RCT of blinatumomab became available^21^ and provided effect estimates that were quite close to those from the external comparison group. This finding illustrated that a suitable inference could have been drawn from the observational results and provides an example of how an external comparison group can be applied.

It should be noted that any methodological approach to adjust for selection bias or confounding depends on the availability of data to fully capture disease risk factors that are imbalanced between the comparison groups. Thus, selection of such factors must be informed by comprehensive models that capture the etiology of the study outcome, the pathways of how persons ended up in one or the other comparison group and the processes that generated the data that are being used. Both lack of data capture, as well as differences in method of data capture, will lead to incompletely balanced groups and may create biased results. This implies that some or many RWD sources may not be suitable to form comparison groups for a given long‐term extension trial. Comprehensive understanding of the limitations of each data source is critical to make such assessments.

The potential for increased sharing of patient level clinical trial data derived from thousands of RCTs may afford an opportunity to better define external comparison groups.^22^, ^23^ Whether the data are generated from placebo or active agent treatment arms of RCTs, the extensive details captured in these data sets may contribute to a reduced selection or information bias, especially when combined with the methods described above. Nonetheless, limitations will still exist even with these detailed data sources (eg, the often limited duration of drug exposure, the non‐contemporaneous nature of the RCT data, or the restrictive inclusion/exclusion criteria used for each trial) which suggest the need for multiple approaches to the construction of external control groups.

Breakout #3 illustrates the value of considering an external control from the outset or at least before a signal arises and what might happen without establishing an external control group in advance of signals being identified. Once a signal arises, there are additional challenges that may be difficult to overcome. An alternative is to monitor and have a plan to add an external control immediately if a signal emerges. A well‐considered external control group, planned and agreed with FDA in advance, could have been beneficial by reducing uncertainty regarding the expected incidence of SIB.

### 2.3 Information bias

A primary design consideration for creating an external comparison cohort derived from real world data sources is to reduce information bias and to quantify potential remaining bias as part of the sensitivity analyses, limitations and generalizability of the study results. To this end, an understanding of the ascertainment and recording practices of the external cohort data source for exposure, outcome and covariate variables is important in order to determine the extent to which practices are similar to those for the trial participants to which comparisons will be made. Striving for similarity in the extent and quality of capture of information between the external control and the clinical trial at the design phase can reduce bias. Following this transparent process is consistent with addressing the criteria of “exchangeability” between a single arm trial or uncontrolled LTE and an external control group proposed in order to reduce information bias and draw a valid comparison.^25^

### 2.4 Exposure measures and duration of follow‐up

While trials are usually characterized by good adherence to randomized exposure assignments, both prescribing decisions and patient decisions to use a particular medication can frequently change as information and alternatives evolve in real‐world practice. Misclassification of exposure in the external control group, may result in bias of effect measures either towards or away from the null.^26^ Decreasing the probability of misclassification of exposure in external control arms assembled from RWD is therefore a design priority. A recommended first step is to evaluate the routine care for the target patient population in the proposed external control group source and to assess how closely it approximates the clinical treatment practice used at trial sites. Regional or health system variations in standard treatment practices or formularies can produce heterogeneity across sites. More than one control group may be needed to address this by providing at least one comparator group that exhibits less heterogeneity, and this should be discussed a priori with regulatory authorities if the analysis is to be part of a formal regulatory submission.

Assuring the comparability of exposure can be accomplished through feasibility assessments that examine patterns of use, such as the mean/median and distribution of continuous exposure to treatment for the exposure groups of interest overall and also restricted to patients who fulfil the selection criteria of the trial sample to confirm that the study could accomplish the stated goal before proceeding to any effect estimation. In addition, there are several points to consider to reduce misclassification of exposure in RWD^27^ such as requiring more than one dispensing or prescription written for the comparison treatment or defining exposure on an as‐treated basis can decrease the probability of misclassifying nonexposed patients as exposed; and assessing the extent of cash payment for treatment outside of prescription insurance benefits (discount cards or over the counter) that do not generate a claim to reduce the probability of misclassifying exposed patients as unexposed.

**FIGURE 1:**
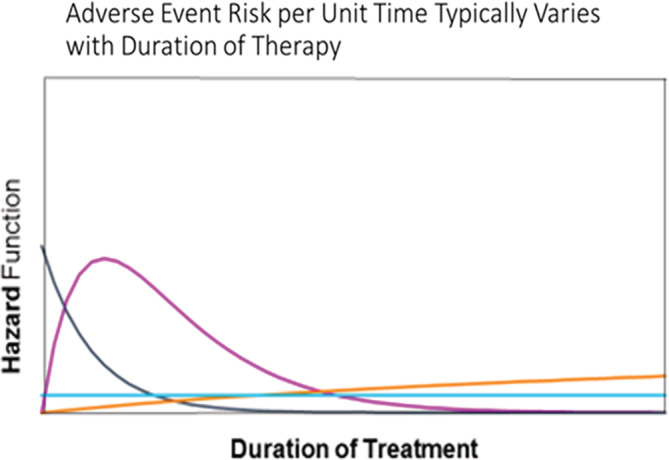
Treatment effects may vary according to time since start of treatment. The light blue line indicates a constant risk of adverse event following treatment start, while the orange line represents a gradually increasing risk with time since treatment start. The dark blue and pink lines correspond to risks that are high early after treatment start and decline over time [Colour figure can be viewed at wileyonlinelibrary.com]

A further consideration is assessing duration of exposure in the external comparator, including the accuracy of start and end dates for treatment. Adequate follow‐up in the external comparator group given the expected outcome incidence can be determined in order to compare to the length of follow‐up in the trial population and provide an effect estimate with suitable statistical power. Beyond duration of therapy, adherence to treatment will need to be evaluated carefully, since medication use characteristics could differ substantially between trial patients and those in the external comparator group. If the study is designed to assess longer term effectiveness and safety measures, duration of follow‐up will be a critical design feature both in terms of quantity of follow‐up, as well as the validity of the exposure recording over time. An external comparison group for an uncontrolled LTE may group exposure periods in a way that corresponds to the biologic plausibility of the effects. Defining exposure on an intent‐to‐treat would be difficult to operationalize the same way in the trial and external control group, and the analysis should recognize this limitation. It is important to consider mechanism of action including known induction periods before a treatment effect could be observed in defining the study comparisons (see Figure 1). For acute effects of treatment, as‐treated analyses may more closely match the mechanism of effect. Outcomes with extended latency periods may be more appropriately analyzed using a variation on intent‐to‐treat. It should be noted in this context that the need to use ITT to preserve randomization at the risk of diluting the observed treatment effect due to exposure misclassification does not apply to observational designs. Instead treatment discontinuation or switching can be considered (in both the external comparison and the single treatment or LTE group). For example, discontinuation might be particularly relevant in assessment of treatment safety (if discontinuation is prompted by an adverse event). In non‐inferiority scenarios, discontinuation of the experimental treatment could lead to crossover to the comparator, which would tend to equalize event rates between the two cohorts. Such informative censoring scenarios can be addressed through use of inverse probability of censoring weighting (IPCW).^28^

Breakout Box #2. **Blinatumomab and external comparison group for a single‐arm trial^18^, ^19^**

Gokbuget and colleagues examined the effectiveness of blinatumomab as a treatment for acute lymphoblastic leukemia (ALL) in adult patients with relapsed/refractory (R/R) ALL, defined as patients with Ph‐negative, B‐precursor ALL who had early relapse (≤ 1 year) or relapse that occurred more than 1 year after treatment where salvage treatment had failed. The authors used results from 189 patients in a single‐arm trial of blinatumomab conducted at multiple sites across Europe and the United States in the years 2010‐2014 for whom complete response (CR) and overall survival (OS) outcomes were recorded. For comparison, the authors used historical data from the years preceding the trial (1990‐2013) from adult patients with R/R ALL collected by six national study groups and five large treatment centers that subsequently participated in the single‐arm trial. These historical data also included the outcomes of CR and OS for a subset of the patients.

Several approaches were used to address differences between patients in the historical data and patients in the clinical trial. As a first approach to produce similarity between trial participants and historical controls, selection criteria were applied to the historical controls that were similar to the selection criteria for the single arm trial: (a) adult patients with Ph‐negative, B‐precursor R/R ALL (b) age 15+ at initial ALL diagnosis (c) initial ALL diagnosis 1990 or later (d) no CNS involvement at time of relapse (e) no isolated extramedullary relapse (f) no previous treatment with blinatumomab. However, even after application of these criteria, substantial differences were noted between the single‐arm trial and the historical control group. The historical comparison group was younger and had more primary refractory ALL along with less use of allogeneic hematopoietic stem cell transplantation and a longer time from initial diagnosis. These remaining differences were addressed using two different analytic approaches. The first approach, standardization, was conducted by stratifying the cohorts by age, stem cell transplantation, and salvage therapy and re‐weighting the historical cohort to match the distribution of these strata in the blinatumomab cohort. The second approach was to develop an exposure propensity score comprised of eight variables and then use the propensity score to re‐weight the groups by Inverse Probability of Treatment Weighting (IPTW). The standardization approach produced results that suggested the blinatumomab treated patients had higher CR and longer OS overall, and within each of the subgroups defined by important prognostic variables. The propensity score‐weighted comparison provided similar results, with blinatumomab treated patients exhibiting higher CR and longer OS.

The addition of external controls to the single arm trial provided better understanding of the results in relation to the heterogeneity of patients with R/R ALL and helped support assumptions of the single‐arm trial, particularly the lower limit of the primary endpoint.

External controls used: Registry data.

Influence on decision‐making: Approval based on trial, and external controls were to verify the study assumption.

Breakout Box #3. **Missed opportunity: Lack of a pre‐specified external comparator for an uncontrolled long‐term extension of clinical trial^24^**

The purpose of this example is to present a situation in which an appropriate external control group might have provided the necessary context to inform labeling of a newly‐approved product.

Brodalumab is indicated in patients with moderate‐severe plaque psoriasis. It binds to the IL‐17 receptor. During development, the clinical trial designs included placebo controlled (12 weeks) periods and then comparative data through 52 weeks. This was followed by a long‐term uncontrolled extension study. The phase 3 data demonstrated that brodalumab is efficacious.

Suicidal ideation and behavior (SIB) were infrequent in randomized, placebo‐controlled 12‐week period in the studies in psoriasis. There were no events in either the placebo groups (N = 879) or the ustekinumab group (N = 613), and there was one event (1/3066 = 0.03%) in the brodalumab group (N = 3066). In the 16‐week psoriatic arthritis trial, ascertainment of SIB was changed from relying on investigator‐ and subject‐reported events to prospective ascertainment using the e‐CSSRS (electronic version of the Columbia Suicide Severity Rating Scale). Despite this change, results were similar to those in the psoriasis studies. There were no events in the placebo group (N = 320), compared with one event in the brodalumab group (1/639 = 0.2%).

After 52 weeks of follow‐up in the psoriasis trials, there was still no imbalance in SIB between the treatment groups. There were 3 events in the ustekinumab group (N = 613, 504 person‐years, rate = 0.60 events per 100 person‐years) and 7 events in the brodalumab group (N = 4019, 3546 person‐years, rate = 0.20 per 100 person‐years). The 7 events in the brodalumab group included 2 completed suicides, one of which was adjudicated as a possible unintentional overdose.

Using the e‐CSSRS appeared to increase the estimated risks in the longer‐term follow‐up in the psoriasis trials. The rate of 0.20 SIB events per 100 person‐years at 52 weeks (based on the 7 events) in the brodalumab group, increased in the long‐term, uncontrolled follow‐up study (34 events, N = 4464, 9162 person‐years, rate = 0.37 per 100 person‐years). Whether that increase was directly attributable to the change in how events were ascertained or to an actual increase in the rate, could not be determined.

In an effort to provide context at the FDA advisory committee meeting, the company presented SIB rates from several other recently‐approved psoriasis treatments, showing that the rates of attempted and completed suicide were similar across all the drugs (allowing for variability due to small sample sizes). In contrast, the FDA presented cumulative rates of completed suicides from 9 psoriasis drugs. The rate of completed suicides for brodalumab was 57.5 per 100 000 person‐years, compared with 14.5 for a pooled estimate based on several (but not all) of the other products.

The FDA's Division of Psychiatric Products reviewed the data and drew several conclusions relevant to potential utility of an external control group.

1. The 12‐week placebo‐controlled data from 3 phase 3 psoriasis trials for brodalumab showed no significant elevation of SIB for brodalumab compared with placebo.
2. No definitive conclusions could be drawn about the relationship between brodalumab and SIB elevation. The data were considered inadequate due to lack of internal control and lack of pre‐specified analysis plan including external controls.

The FDA approval of brodalumab included a boxed warning for SIB, and it is unknown if use of a contemporaneous external control group would have produced a different result.

External controls used: External RCT data.

Influence on decision‐making: Uncertain.

Breakout Box #4. **Limitations of an external comparison group^32^**

A recent example to consider here is Selinexor KS‐50039. The sponsor, Karyopharm, submitted as part of the new drug application (NDA) an external control cohort from Flatiron Health oncology EMR data to serve as a comparison group to a single arm trial for the outcome of overall survival in patients with penta‐ exposed, triple‐class refractory multiple myeloma. The FDA's review of the evidence highlighted the need for comparability between the populations at baseline; for the Flatiron EMR data this meant restricting to the patients in whom the baseline clinical prognostic variables were measured and available for analysis. The sponsor reanalyzed the data only retaining patients with complete data. The initial analysis submitted showed a significant benefit relative to the matched external control data, however, after restricting the analysis to patients with complete data and adjusting for key baseline factors and eliminating immortal time bias in the definition of the exposure, the sample size was no longer large enough for robust estimation.

TABLE 3 Results from Karyopharm FDA briefing book

**Table:** 

	Initial Analysis (unadjusted)		Revised Adjusted Analysis (restricted to complete matched data)	
	FHAD* (N = 64)	STORM** (N = 122)	FHAD (N = 13)	STORM (N = 64)
Median OS, mo. (95% CI)	3.7 (2.6, 7.1)	9.5 (7.3, 11.9)	12.6 (0.7, 12.6)	10.4 (6.3, NE)
HR (95% CI)	0.41 (0.26, 0.65)		0.63 (0.25, 1.58)	
*P*‐value	.0001		.33

*Flatiron Health Analytic Database (FHAD).

** KCP‐330‐012 (STORM) single arm trial of selinexor + dexamethasone.

External controls used: Oncology EMR data.

Influence on decision‐making: Well‐considered external controls and early engagement on analysis plan with feasibility assessment might have helped the situation by reducing the uncertainty regarding expected occurrence of the outcome.

The completeness of the exposure assessment, especially in the case of combination regimens in “standard of care” comparators, needs to be documented and may include confirming the pharmacy coverage benefits throughout the study period and patient‐level linkage to multiple data sources when possible (eg, outpatient pharmacy claims, procedure codes for infused/injected medicines, health records of prescriptions). When the experimental medication is or becomes available within the external control group, definitions of comparator exposure will need to account for potential cross‐over to experimental exposure or censor patients when that occurs to reduce misclassification of exposure. Remotely‐captured adherence measures (eg, patient‐reported diary, smart devices), when available, can further reduce misclassification for prospectively collected studies (eg, ongoing disease registries) and can be compared to more typical clinical trial data. Certain treatment features, such as newness, expense, insurance coverage, route of administration (eg, infused vs oral) may reduce potential for exposure misclassification.

### 2.5 Outcome measures and potential for loss to follow‐up

Bias can occur if the RWD comparators' outcome assessments differ systematically from trial patients in terms of measurement frequency, method of ascertainment, or definitions. These potential differences are important to both assess and describe. The primary study outcome may dictate which potential data sources are suitable, and those that routinely capture the outcome in a systematic way may be preferred over those that do not.

In the case of oncology trials using EHR‐based external control cohorts, progression‐free survival can be estimated, with larger but not insurmountable problems in estimating overall survival, even in specialist oncology EMR.^29^ Assessment of safety outcomes treated by someone other than the oncologist might be less likely to be identified in oncology clinic‐based EHRs than in claims, which would identify any billable events, including hospitalizations. In general, loss to follow‐up in RWD may be a concern due to change in the patient's health insurance enrollment (claims) or in EHR data due to change to a provider who is outside of the network (eg, referral to a different specialist facility or participation in an RCT) while follow‐up in a trial or registry is agnostic to insurance or provider system but oftentimes limited to the time point that has been deemed sufficient to assess the primary endpoint (which in turn might be different from the endpoint needed for the external comparison study at hand). Validation of outcome definitions in RWD to demonstrate expected impact of outcome misclassification on effect estimates is recommended before moving on to making causal inferences. Moreover, in the initial feasibility assessment evaluations of presence and frequency of assessments for outcomes are appropriate to understand whether differential capture of outcomes between the study arms can be expected.

Assessments of RWD‐based covariates at baseline in the external control group potentially may be more complete than in a clinical trial population, particularly if the data source is claims and/or EHR data, because the RWD‐based information would not be limited to typical CRF questions asked by the nurse or patient self‐report, which tend to report serious or recent events. Alternatively, if specific safety issues are expected, the clinical trial may use a more comprehensive ascertainment protocol providing more sensitive and detailed outcomes ascertainment than RWD. The main covariates of interest would be those that were important to selection of the external cohort (ie, to reproduce the inclusion/exclusion criteria for the trial) and/or to use for adjustment of potential confounding to balance out the differences in baseline risk for the clinical trial and external comparator cohort populations (eg, to build the propensity score model). Further covariates that might be important stratification factors for the primary analysis or sensitivity analyses (eg, prognostic/severity markers), along with their comparability in measurement, would need to be assessed. A limitation of RWD sources could be unmeasured covariates that are associated with the outcome risk, such as lifestyle characteristics (obesity, smoking, etc.), which could bias estimates relative to a more selected and motivated population in an RCT. Importantly, misclassified confounders and especially misclassification that is differential across comparison groups can introduce bias, with a solid understanding of the data generating processes helpful to identify such biases. Probabilistic sensitivity analyses that predict the degree of biases given varying misclassification scenarios can be used to quantity the robustness of study results.

### 2.6 Missing data

As part of the feasibility assessment to inform the decision of which external data sources would be fit‐for‐purpose to create an appropriate comparator cohort for a single arm or LTE clinical trial, characterization of the frequency and completeness of crucial data fields is an important step. One of the main concerns to address is the extent of missingness in the external control cohort, particularly when the data originate from routine clinical practice. The first step is to understand if (a) data elements were collected but not available in the data set (eg, a longitudinal EMR record linking patient's diagnosis, procedure, and medication codes from specialty oncology practice may not capture all baseline comorbid conditions/treatments provided by other HCPs including primary care, although such information may be extractable from notes using machine‐learning or natural language processing tools or from pursuit of linkage to additional data, such as manual medical record review), (b) if they are missing completely at random (MCAR), for example, busy clinicians did not record or a practice dropped out of the EMR, or (c) if they are systematically missing (eg, patients who seek more care are often feeling worse so missing data could indicate a better prognosis or missing data could result from less rigorous follow‐up and indicate poorer quality of care).^30^ For example, prognosis of at least 4 months survival at start of treatment as assessed by the study investigators among patients with relapsed refractory multiple myeloma, which was used as one of the selinexor trial (Breakout Box #4) inclusion criteria, could not be captured in the EMR database used to develop the external comparison group, raising concerns about differential mortality risk among the groups.

Assessing the extent of missingness and then the extent to which it varies by measured factors (eg, recording of comorbid conditions varies by HCP/center, or weight recording varies by patient age) is important in clinical trials and in real‐world data and is a first step in understanding how to manage the missing data with analytic strategies or at the design level by selecting a comparator from historical clinical trial data. Approaches for dealing with missing data in EHR data also have been investigated.^31^

### 2.7 Complete case analysis

An option to manage missing data is to restrict the analysis to the patients without any missing data in order to produce direct comparability between the measured variables in the RCT and external comparator group. This method can introduce bias depending on the reason for missingness, as well as large reductions in sample size, which could lead to lower external validity, a lack of precision in the effect estimate and ultimately inconclusive results.

### 2.8 Imputation

An alternative approach to restricting to the patients with complete data capture, which can preserve sample size and avoid bias if missing is not completely at random, is to retain patients with a minimum level of completeness and impute missing values to create a larger data set on which to run effect estimation models. The usual recommendation for imputation is the technique of multiple imputation which is standard procedure available in many statistical software packages under the assumption that data are missing at random (MAR). Missing values are substituted with some form of “predicted” value, based on a model, but random error is introduced. The process is repeated multiple times (at least 5 and up to the number of % missing) to capture the variability inherent to the imputation process. That variability can affect not only the coefficients for the variables with missing data, but also for all variables included in the model.^33^, ^34^ Single imputation tends to overstate the level of precision and is not recommended, especially with real world data which would not be expected to meet the assumptions of missing completely at random (MCAR).^35^ It is important to clearly describe methods used for imputation in order to facilitate replication in other data sources.

## 3 DISCUSSION

The validity of inferences drawn from a single arm trial or uncontrolled LTE largely depends on using an external control group in a way that addresses both the selection bias and information bias that are likely to be present.

Understanding the process that generated the data in both the trial and the external control arm is critical to predict presence of selection and information bias. It will drive the selection of the most appropriate RWD sources and inform the pharmacoepidemiologic design and statistical techniques used to address such biases. As the infrastructure for incorporating clinical trials becomes more routinely integrated into routine healthcare delivery settings, the quality of RWD resulting from these practices is expected to increase.

### 3.1 Recommendations

We advise consideration of the merits of an external control group(s) for single arm trials or long‐term extensions relative to no control group on aspects of the study, including efficiency, ethics, reduced uncertainty in decision making (by providing an appropriate comparator group), and potential accelerated access to patients with unmet needs. Further, we suggest starting with an assessment of suitable data sources where the biases can be reasonably addressed. Making an informed decision among available data sources (eg, re‐using historical clinical trial data, existing registries, curated integrated EHR) should be based on trade‐offs between the consistency and compatibility of data capture with the clinical study for key variables (clinical characteristics, exposure, and outcomes) for the specific research question to be addressed. These will facilitate addressing questions of both selection and information bias in the comparison.

Focusing on sound pharmacoepidemiology methods to reduce bias in developing the external comparison group both in design and analysis phases will support causal inference. In the case of a long‐term extension, characterizing new user follow‐up at the time of cross‐over is essential to making inferences. Investigators should thoroughly describe the limitations involved in the comparisons, including potential for bias and should attempt to quantify bias where feasible.

In the scenario of generating evidence to inform regulatory decision making, for example, supplemental application for a new indication, it is advised to discuss the plan for an external comparison group with the regulatory authority early in the process.^36^ When there are no prior studies to serve as references, conduct validation studies, as feasible, to increase confidence in the measurements arising from RWD source (specific sub‐population of interest) and the RCT.

There may be situations where external control data are sparse but still useful for providing context in the evaluation of safety or effectiveness.

## 4 CONCLUSIONS

Assuming appropriate data, design and analytical techniques, an external control group can provide appropriate context for a single arm trial or long‐term extension of an RCT to facilitate causal inferences on treatment benefit or risk leading to reduced uncertainty in decision making by health authorities, healthcare practitioners, and patients.
